# Reference Intervals of Spot Urine Creatinine-to-Osmolality Ratio as a Surrogate of Urinary Creatinine Excretion Rate

**DOI:** 10.1155/2022/3549047

**Published:** 2022-07-25

**Authors:** Shih-Ping Hsu, Chiang-Ting Chien

**Affiliations:** ^1^Department of Internal Medicine, Far Eastern Memorial Hospital, New Taipei, Taiwan; ^2^School of Life Science, National Taiwan Normal University, Taipei, Taiwan; ^3^College of Medicine, National Taiwan University, Taipei, Taiwan

## Abstract

A spot urine creatinine-to-osmolality ratio (sUCr/Osm) is proposed as a surrogate of the urinary excretion rate of creatinine (Cr) and convenient for forecasting serum Cr (SCr) trends. The lower the sUCr/Osm, the lower the excreted Cr amount accompanied by per unit of osmoles, the higher the risk of Cr accumulation. For exploring the reference intervals of sUCr/Osm in general adults, a cross-sectional analysis was performed on a subset of data from the National Health and Nutrition Examination Survey (NHANES) 2011–2012. Of the eligible 3,316 adults aged 18.0 to 79.9 years, the age (mean ± SD) was 45.2 ± 17.2 years old, women was 45.02%, body weight (BW) was 76.1 ± 14.5 kg, and African Americans was 23.6%. Blood urea nitrogen (BUN) was 12.6 ± 4.7 mg/dL; SCr was 0.89 ± 0.34 mg/dL. As spot urine Cr and osmolality were 127.1 ± 84.0 mg/dl and 649 ± 266 mOsm/kg, respectively, sUCr/Osm was 0.19 ± 0.08. With adjustment of factors related to personal urinary excretion of Cr and osmoles by multivariable regression analysis, the estimated sUCr/Osm (esUCr/Osm) for an individual was 0.153 × (age in year)^−0.070^ × (BW in kg)^0.283^ × 1.244 [if African American] × (BUN in mg/dL)^−0.310^ × (SCr in mg/dL)^0.681^. The index of sUCr/Osm to personalized esUCr/Osm was 1.05 ± 0.39. When only low urinary excretion of Cr is likely to be of clinical concern, further analysis showed 157 individuals (4.7%, outside the 5th percentile) had their original sUCr/Osm < 0.08; 157 had the sUCr/Osm indexed for personalized esUCr/Osm < 0.50.

## 1. Introduction

Renal clearance rate of creatinine (Cr), the ratio of urinary excretion rate to plasma concentration in terms of Cr, has been conventionally regarded as a clinical surrogate of glomerular filtration rate (GFR). In an individual with homeostasis of Cr, the amount of production or renal excretion load is supposed to be steady and equal to the amount of urinary excretion during a certain period. On the assumption that the urinary excretion rate of Cr in a stable individual is steady, serum Cr (SCr) related inversely to GFR is consequently steady. Thus, SCr-based definitions of renal excretion function are dominantly in practice. Nowadays, widely accepted estimated GFR (eGFR) formulas, such as MDRD equation [1], MDRD-EPI Creatinine equation [2], and MDRD-EPI Creatinine-Cystatin equation [3], are all developed for conditions with assumptively steady SCr levels. However, for a situation with ever-changing SCr levels as in acute kidney injury (AKI), few suitable manners for the evaluation of instant renal excretion function have been proposed [4, 5].

A spot urine creatinine-to-osmolality ratio (sUCr/Osm) is the ratio of the Cr concentration to osmolality in a urine sample, independent of the actual urine volume, urination interval, and concurrent SCr level. The daily urinary excretion of Cr and renal osmolar loads in an individual is presumed stable and estimable. The endogenous Cr generation, almost excreted in the urine, is largely a function of muscle mass, affected by age, sex, race, and body weight (BW) [6, 7]. When an individual has usual diet and no excessive catabolic loads due to diseases or medication use, his or her daily renal load and urinary excretion of osmoles are also assumed stable [8, 9]. These fundamental rationales have been applied in the studies about spot urine albumin/protein to Cr ratio [10–12] and albumin/protein to osmolality ratio [13, 14]. In addition, the urinary excretion rates of Cr and osmoles are in concordance with each other during a typical day [15]. Then, sUCr/Osm is a plausible surrogate of urinary Cr excretion rate. The lower the sUCr/Osm, the lower the excreted Cr amount accompanied by per unit of osmoles, the higher the risk of Cr accumulation in the body.

Reference intervals, based on the results that are seen in 95% of the healthy reference population, are the most common decision support tool used for the interpretation of numerical pathology reports before clinical decision limits are defined in further validation trials [16–18]. When an individual's sUCr/Osm is lower than the lower reference limits, they may be at the highest risk of acute accumulation of Cr in the body, increasing SCr levels, and even meeting the SCr criteria of AKI [19].

Therefore, to provide a cornerstone for further validation trials in various clinical conditions, in this study, we first tried to explore the reference intervals of sUCr/Osm in ordinary adults in stable condition. Besides, the physiological rationale of sUCr/Osm as a surrogate of the instant urinary excretion rate of Cr is to be elaborated in Discussion.

## 2. Methods

### 2.1. Study Population

Conducted by the U.S. National Center for Health Statistics, NHANES is a continuous nationally representative survey. However, urinary Cr and osmolality were measured only for individuals enrolled in NHANES 2009-2010 and 2011-2012. In 2011-2012, 13,431 individuals were selected. Of those selected, 9,756 completed the interview and 9,338 were examined. The data of the 9,756 participants in the NHANES 2011–2012 were first openly published in September 2013 and last revised in January 2015 (https://wwwn.cdc.gov/Nchs/Nhanes) [20]. All definitions of the following parameters of demographics, comorbidities, and medication use, as well as details in measurement of blood biochemistry, urine Cr, and osmolality, were summarized from the open-source documents available on the same website.

In the present secondary analysis study to investigate the reference values of sUCr/Osm in general adults, we restricted the analysis to participants between the ages of 18 to 79.9 years, considering that individuals aged 80 and over were all coded as 80 in the original data set of NHANES 2011–2012. To exclude extraordinary or diseased conditions known with interference with the urinary excretion load of Cr and osmoles (such as extreme body mass, malnutrition, severe liver dysfunction, rhabdomyolysis, abnormal serum osmolality, dysnatremia, and dyskalemia), further filtration was performed with reasonable ranges of the following relevant anthropometrics and laboratory tests: body mass index (BMI, kg/m^2^), 18.5-34.9 (World Health Organization [21] and Centers for Disease Control and Prevention [22]: normal weight to obesity class I); albumin (g/dL), ≥3.5 (35 in g/L); glutamic oxaloacetic transaminase (GOT, IU/L) and glutamic pyruvic transaminase (GPT, IU/L), ≤120; total bilirubin (mg/dL), ≤2 (34.2 *μ*mol/L); creatine phosphokinase (CPK, IU/L), <1,500; serum osmolality (mOsm/kg), 270-299; serum Na (meq/L), 135-144; and serum K (meq/L), 3.5-5.5. The final data set of 3,316 eligible participants was used for analysis in this cross-sectional study.

The NHANES 2011–2012 was approved by the institutional review board of the National Center for Health Statistics. Oral and written informed consent from all participants was obtained by the National Center for Health Statistics. According to the U.S. Federal Policy for the Protection of Human Subjects (45 CFR 46), the secondary analysis of publicly available, existing deidentified data, such as NHANES 2011–2012, does not fall within the regulatory definition of research involving human subjects and not require Committee for Protection of Human Subjects (CPHS) review. We declare all methods of data processing and result interpretation in the present secondary analysis study have been performed in accordance with the Declaration of Helsinki.

### 2.2. Demographics, Comorbidities, and Medication Use

Demographic variables including age, sex, and race were collected during the interview. The body measure examination was performed with identical equipment.

Hypertension was defined as a self-reported physician diagnosis, use of antihypertensive medication, mean systolic blood pressure > 140 mmHg, or mean diastolic blood pressure > 90 mmHg. Diabetes mellitus was defined as a self-reported physician diagnosis, use of diabetic medication, or glucose levels ≥ 126 mg/dL (7.0 mmol/L) (fasting 8 hours or more) or ≥200 mg/dL (11.1 mmol/L) (fasting less than 8 hours). Other comorbid medical conditions were based on self-reported personal interview data on a broad range of health conditions.

Information on prescription medications was collected by trained interviewers during the household interview. We explored the use of angiotensin converting enzyme inhibitors (ACEIs), angiotensin II receptor blockers (ARBs), and diuretics, as well as other antihypertensive medications. Diuretic use was defined as participants taking thiazide-like agents, loop-diuretics, or potassium-sparing agents given in monotherapy or in combination.

### 2.3. Measurement of Blood Biochemistry, Urine Creatinine, and Osmolality

In NHANES 2011–2012, serum specimens were processed, stored, and shipped to the Collaborative Laboratory Services for analysis. Detailed specimen collection and processing instructions were available in the NHANES Laboratory/Medical Technologists Procedures Manual (LPM). Vials were stored under appropriate frozen (-30°C) conditions until they were shipped to the National Center for Environmental Health for testing. SCr was measured by the Jaffé rate method (kinetic alkaline picrate) using a Beckman Coulter UniCel® DxC800 Synchron at the Collaborative Laboratory Services at Ottumwa, Iowa, in 2011–2012.

For urine Cr analysis, spot or timed urine samples were stored at 2–8°C until analysis within 36 hours of receipt in the laboratory. Urine Cr was measured by an enzymatic (creatinase) method with Roche/Hitachi Modular P Chemistry Analyzer. For urine osmolality, spot or timed samples were analyzed directly at the mobile examination center within 4 hours of collection. Urine osmolality was measured by the freezing point depression method with Osmette II, Model 5005 Automatic Osmometer.

### 2.4. Statistical Analysis

Statistical analyses of the original data in NHANES 2011–2012 were performed using the sample survey commands in STATA version 12.0 statistical software (StataCorp LP, College Station, Texas). The data in the present study were processed, filtered, and analyzed with IBM® SPSS® Statistics 22.0 (New York, United States).

Unless otherwise stated, continuous variables are presented as mean ± standard deviation (SD) and categorical variables as a number (%) for each item. As indicated, the differences within binary or categorial variables were compared using the Student *t*-test, the Chi-square test, or the one-way analysis of variance (ANOVA) along with the Bonferroni method as the post hoc test. For exploring factors related to spot urine Cr concentration (sUCr), spot urine osmolality (sUOsm), and sUCr/Osm, univariable and then multivariable regression models were tested with demographic and laboratory variables as independent variables. Since the logarithmic models were found to fit better to the univariable regression models, the data of all variables were processed first with logarithmic transformation for further linear regression analysis. Unless specifically stated, a *P* level < 0.05 for two-tailed tests was considered statistically significant.

## 3. Results

### 3.1. Demographics, Comorbidities, and Medication Use

Of the 3,316 eligible adults without extraordinary conditions predefined in Methods, the age was 45.2 ± 17.2 years old. Women were 1,493 (45.02%). The BW was 76.1 ± 14.5 kg. Non-Hispanic Blacks (African Americans) were 781 (23.6%). Hypertension was noted in 834 (25.1%); diabetes mellitus was noted in 392 (11.8%). Of them, 477 (14.4%) individuals took angiotensin-converting-enzyme inhibitors (ACEIs) or angiotensin II receptor blockers (ARBs) and 280 (8.4%) took diuretics. Other details are shown in Table 1.

### 3.2. Blood Biochemistry and Urine Profile

Of the 3,316 individuals, blood urea nitrogen (BUN) was 12.6 ± 4.7 mg/dL; SCr was 0.89 ± 0.34 mg/dL. In spot urine samples, sUCr was 127 ± 84 mg/dl; sUOsm was 649 ± 266 mOsm/kg; urine albumin-to-creatinine ratio (UACR) was 33.0 ± 323.0 mg/g. Thus, their sUCr/Osm was 0.19 ± 0.08, with the median as 0.1761. Other details are shown in Table 2.

### 3.3. Factors Related to Spot Urine Profile

To investigate factors related to the spot urine profile, the binary/categorical variables were analyzed first. The women had lower sUCr, sUOsm, and sUCr/Osm. African Americans had higher sUCr, sUOsm, and sUCr/Osm than the other races. Individuals with diabetes mellitus, cancer, or diuretics use had lower sUCr. On the other hand, those with hypertension, diabetes, cerebrovascular accident, cancer, or use of any kind of antihypertensives had lower sUOsm. As to sUCr/Osm, only the individuals taking *β*-adrenergic blockers, calcium channel blockers (CCBs), or other antihypertensive agents had higher values. The details of comparisons are shown in Tables 3–5.

Then, the continuous variables were analyzed with univariate regression methods. The regression analysis was performed on natural logarithm-transformed data, for they fit better for further linear regression models. We found sUCr was correlated negatively with age, MDRD eGFR, serum albumin, phosphate, and UACR and positively with BW, BMI, BUN, SCr, GPT, total bilirubin, uric acid, CPK, serum osmolality, Na, and sUOsm. Similarly, sUOsm was correlated negatively with age, serum albumin, calcium, phosphate, and UACR and positively with BW, BMI, BUN, SCr, MDRD eGFR, GPT, uric acid, CPK, glucose, serum osmolality, Na, and sUCr. As to sUCr/Osm, it was correlated negatively with age, BUN, MDRD eGFR, serum phosphate, and UACR and positively with BW, BMI, SCr, GOT, total bilirubin, uric acid, CPK, Na, sUCr, and sUOsm. (Table 6).

For exclusion of possible confounding effects, the abovementioned binary variables with significant differences and continuous variables with significant correlations were adopted as independent variables in the multivariable regression analysis with the spot urine profile as dependent variables. Of note, BW, rather than BMI, was adopted as the representative variable of body size, considering the effect sizes estimated by the standardized coefficients (0.247 vs. 0.109). Conventionally, age, sex, BW, and race are considered to be the main factors related to endogenous Cr generation [6, 7]. When age, sex, BW, and race were forced-in variables in the multivariable regression model, sUCr was correlated negatively with age, BUN, and glucose and positively with female sex, BW, African American race, use of CCBs, SCr, total bilirubin, uric acid, and sUOsm. On the other hand, sUOsm was correlated negatively with age, female sex, use of *β*-adrenergic blockers, SCr, serum albumin, uric acid, and phosphate and positively with BUN, glucose, serum osmolality, and sUCr. As to sUCr/Osm, it was correlated negatively with age, BUN, presence of diabetes mellitus, and serum calcium and positively with BW, African American race, use of CCBs, SCr, total bilirubin, and uric acid (Table 7). Furthermore, when only variables with standardized coefficients > 0.10 and *P* values < 0.001 were counted in using Goldilocks balance between the number of predictors and the gain in adjusted R-square, the estimated sUCr/Osm (esUCr/Osm) for an individual = 0.153 × (age in year)^−0.070^ × (BW in kg)^0.283^ × 1.244 [if African American] × (BUN in mg/dL)^−0.310^ × (SCr in mg/dL)^0.681^, with the adjusted *R* − square = 0.247. Considering sUCr, sUOsm, and sUCr/Osm varied with the abovementioned personal features, it is reasonable that interpersonal comparisons about sUCr/Osm should include some adjustment for the personalized factors as indicated, such as personalized esUCr/Osm. Therefore, the adjustment method was to index sUCr/Osm to personalized esUCr/Osm. The sUCr/Osm indexed for personalized esUCr/Osm was 1.05 ± 0.39, ranging 0.14–4.74, with the median as 1.01.

**Figure 1:**
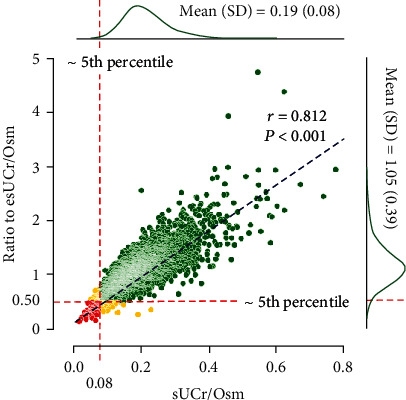
Relationship between spot urine creatinine-to-osmolality ratio (sUCr/Osm) and the values indexed for personalized estimated sUCr/Osm (ratio to esUCr/Osm). green dots, ≥ both 5th percentiles; yellow dots, only < either 5th percentile; red dots, < both 5th percentiles.

Conventionally, the reference interval for a given test is based on the results that are seen in 95% of the reference population [16, 17]. Since only low renal excretion of Cr is likely to be of clinical concern, a left-sided 95% reference interval of sUCr/Osm and the use of the 5th percentile as a one-sided lower reference limit make the most sense. Therefore, further analysis revealed that 157 individuals (4.7%, less than the 5th percentile) had their original absolute values of sUCr/Osm < 0.08; 157 (4.7%) had the sUCr/Osm indexed for personalized esUCr/Osm < 0.50. Of the 111 (3.3%) participants, the sUCr/Osm were simultaneously lower than both the lower reference limits of the above two one-sided reference intervals. A visual summary is shown as Figure 1.

## 4. Discussion

From the subset of data extracted from the original NHANES 2011-2012, we found the original sUCr/Osm was 0.19 ± 0.08 and the value indexed for personalized esUCr/Osm was 1.05 ± 0.39. Approximately, 4.7% ordinary adults had their sUCr/Osm less than 0.08, whereas 4.7% ordinary adults had the values indexed for personalized esUCr/Osm less than 0.50. Besides, as explored in the multivariable regression model, age and BUN were the major (with the standardized coefficients > 0.10), negatively correlated factors of sUCr/Osm, whereas BW, African American race, and SCr were the major, positively correlated factors (Table 7).

Although few studies have reported the relationship between Cr and osmolality in spot urine samples [23–25], it is a novel concept regarding sUCr/Osm as a surrogate of instant urinary Cr excretion rate. Now, the physiological rationale is further elaborated as follows.

Spot urine Cr concentration, sUCr, is the amount of Cr excreted in the urine during an uncertain urination interval divided by the corresponding volume, even if there is residual urine. It is also independent of the concurrent SCr levels. Theoretically, for keeping Cr homeostasis in the body, the total amount of urinary Cr excretion, including glomerular filtration and tubular secretion, must balance the estimated load. The daily load of urinary Cr excretion in an individual with stable SCr levels could be estimated by using various equations considering age, sex, BW, body surface area, race, and even serum phosphorus levels [7]. Recently, it has been discovered that urinary excretion of Cr shows circadian changes with a peak in the afternoon and evening [15].

Likewise, sUOsm is the number of osmoles of solute excreted in the urine during an uncertain urination interval divided by the corresponding weight (approximately urine volume). For keeping osmolar homeostasis in a steady state, the amount of daily renal osmolar excretion is tailored to the amount of daily osmolar load minus unregulated extrarenal excretion and loss (mainly in feces and sweat). In general, daily renal osmolar excretion in an adult is responsible for more than 90% of the daily load. In individuals taking a typical Western diet, the daily renal osmolar excretion load is estimated as 600 to 900 mOsm [8]. There are also circadian variations in urinary osmolar excretion rates and approximately in concordance with those about urinary Cr excretion rates [15].

However, neither sUCr nor sUOsm per se is a proper surrogate of urinary excretion function, for there is no information about the actual urination interval and volume.

By contrast, sUCr/Osm is the relative value of excreted Cr amount to osmoles in a urine sample, independent of actual urination intervals and SCr levels. The urinary excretion rates of Cr and osmoles are in concordance with each other during a typical day [15]. In addition, the daily urinary excretion of Cr and renal osmolar load in an individual is presumed stable, with the exception of specific conditions listed in Table 8. Therefore, sUCr/Osm is a plausible surrogate of near real-time urinary Cr excretion rate. The lower the sUCr/Osm, the less the urinary excretion of Cr in proportion to accompanied osmoles, and the higher the possibility of Cr accumulation in the body. No matter if the reference intervals are determined using absolute values or values indexed for personalized esUCr/Osm, an individual's instant urinary Cr excretion can be reasonably assessed. If his or her sUCr/Osm is lower than the lower reference limits, it is inferred the urinary Cr excretion rate has been less than the 5th percentile at the sampling time. Then, they are at the highest risk of acute accumulation of Cr in the body and subsequently increased SCr levels.

On the basis of the present study, the lower reference limits of sUCr/Osm in adults are proposed as 0.08 for the absolute value and 0.50 for the value indexed for personalized esUCr/Osm. As a result, less than 5% people will have their sUCr/Osm lower than the lower reference limits. Furthermore, about 3.3% of people's sUCr/Osm are lower than the above two lower reference limits at the same time.

Although there was a good positive correlation between sUCr and sUOsm, the factors related to sUCr, sUOsm, and sUCr/Osm were not always consistent in their respective correlations (Table 6) First, in the present analysis, we found the older the age, the lower the sUOsm and sUCr/Osm. During normal aging in people, urine concentrating ability is known to reduce [26]. In a previous analysis with the data of 10,769 participants aged 16 years or older in the NHANES 2009–2012 [24], both of sUCr and sUOsm were correlated negatively with the per 10-year increase in age. Second, when women had lower sUCr and sUOsm, the effect of sex differences on sUCr/Osm was eventually nonsignificant after adjustment for other confounding factors, such as age, BW, BUN, and SCr. Third, the higher the BW, the higher the sUCr and sUCr/Osm, in accordance with that the fact daily production of Cr is positively correlated with BW in adults [6]. Although daily renal osmolar load was deemed to be positively correlated with individual BW [9], sUOsm was not found to be correlated with BW in the present study. Fourth, African Americans had not only higher sUCr but also higher sUCr/Osm. It is compatible with the presumption that African Americans have a higher daily urinary excretion load of Cr, as supported by the fact that higher eGFR for African Americans at a given SCr level is consistent with various eGFR equations [1–3]. When BUN, SCr, and their interactions were considered at the same time, the ratio of BUN to SCr (BUN/SCr) was correlated negatively with sUCr as well as sUCr/Osm and positively with sUOsm. Reasonably, the higher the SCr, the higher the sUCr, even after correction for simultaneous urine osmolality. The positive correlation between sUOsm and BUN/SCr is compatible with the fact that increased urine concentration power or tubular reabsorption of UN leads to higher BUN/SCr as in the case of prerenal azotemia. As to the correlations of uric acid with sUCr, sUOsm, and sUCr/Osm, they would be attributed to the status of food intake, intrinsic catabolism, and renal absorption and excretion. For conciseness, other possible minor factors merely related to sUCr or sUOsm, and those for sUCr/Osm but with a *P* value > 0.001 are not to be comprehensively discussed.

There are some limitations in the present study that need to be noted. First, in the original NHANES 2011-2012, there was no information about the major components contributing to urine osmolality such as concentrations of sodium, potassium, and UN in urine. Second, despite using multivariable regression models with adjustment of available confounding variables, the findings in this cross-sectional study only provided associations, not causation, about the relationship between the spot urine profile and the features of demographics, medical conditions, medication use, and laboratory tests. Third, for individuals not within the reasonable ranges regarding relevant anthropometrics and laboratory tests as listed in Methods, the reference intervals or lower reference limits of sUCr/Osm would not be applicable. Fourth, as race is a social construct rather than a biological variable, race adjustment about African Americans has been questioned recently [27]. However, in the present study using the data from NHANES 2011–2012, the actual effects of race differences were still taken into account for data presentation, analysis, and interpretation.

## 5. Conclusions

According to the Kidney Disease: Improving Global Outcomes (KDIGO) definition and staging system guidelines [19], the consensus on AKI definition is as follows: (1) increase in SCr by ≥0.3 mg/dL (≥26.5 *μ*mol/L) within 48 hours or (2) increase in SCr to ≥1.5 times baseline, which is known or presumed to have occurred within the prior seven days, or (3) urine volume < 0.5 mL/kg BW/hour for six hours. Thus, based on the definition, increase in SCr levels or decrease in urine volume, the diagnosis of AKI must be deferred for at least six hours after entry, even in emergency settings. Therefore, to meet the need of noninvasive diagnosis of early kidney injury, biomarkers of tubular injury and/or damage have been extensively investigated [28, 29]. However, acute dysfunction manifesting as increasing SCr levels might occur without tubular damage indicated by those injury biomarkers [29].

As introduced as a physiologically sound surrogate of instant urinary Cr excretion rate, sUCr/Osm would be promising as a practical tool for the assessment of risks of acute accumulation of Cr in an individual originally assumed in stable condition. If the sUCr/Osm is lower than the lower reference limit, 0.08 for absolute values or 0.50 for values indexed for personalized esUCr/Osm, it can be inferred his or her urinary Cr excretion rate at the sampling time would be below the 5th percentile. Then, they may be at the highest risk of acute accumulation of Cr in the body, increasing SCr levels, and meeting the SCr criteria of AKI.

The present study only provided the preliminary concept of sUCr/Osm as a surrogate of instant urinary Cr excretion rate and its reference intervals for ordinary adults in stable condition. There are other clinical situations with various interferences in urinary excretion of Cr and osmoles as listed in Table 8. Therefore, further validation trials are advocated for verification of sUCr/Osm employed for recognition of true AKI risk in various clinical conditions.

## Figures and Tables

**Table 1:** Demographics, comorbidities, and medication use (*N* = 3,316).

	Mean ± SD (range) or number (%)
Age, year	45.2 ± 17.2 (18.0-79.9)
Female sex	1,493 (45.0)
Body weight, kg	76.1 ± 14.5 (39.3-125.7)
Body mass index, kg/m^2^	26.8 ± 4.0 (18.5-34.9)
Races	
Mexican American	361 (10.9)
Other Hispanic	483 (11.6)
Non-Hispanic White	1172 (35.3)
Non-Hispanic Black	781 (23.6)
Non-Hispanic Asian	526 (15.9)
Other races	93 (2.8)
Comorbidities	
Hypertension	834 (25.2)
Diabetes mellitus	392 (11.8)
Coronary artery disease	146 (4.4)
Congestive heart failure	63 (1.9)
Cerebrovascular disease	81 (2.4)
Active liver disease	57 (1.7)
Cancer	213 (6.4)
Medication use	
ACEI/ARB	477 (14.4)
*β*-Adrenergic blocker	255 (7.7)
Calcium channel blocker	209 (6.3)
Diuretics	280 (8.4)
Other antihypertensives	87 (2.6)

Abbreviations: ACEI: angiotensin converting enzyme inhibitor; ARB: angiotensin II receptor blocker; SD: standard deviation.

**Table 2:** Laboratory data (*N* = 3,316).

	Mean ± SD (range)
Blood biochemistry	
Urea nitrogen, mg/dL	12.6 ± 4.7 (2-57)
Creatinine, mg/dL	0.89 ± 0.34 (0.38-7.46)
MDRD eGFR, ml/min/1.73 m^2^	93.2 ± 23.8 (7.4-206.9)
Albumin, g/dL	4.3 ± 0.3 (3.5-5.5)
GOT, IU/L	24.4 ± 8.8 (7-120)
GPT, IU/L	23.6 ± 12.9 (5-116)
Total bilirubin, mg/dL	0.7 ± 0.3 (0.1-2.0)
Uric acid, mg/dL	5.4 ± 1.4 (0.4-11.0)
CPK, IU/L	159 ± 143 (21-1488)
Glucose, mg/dL	98 ± 32 (47-526)
Osmolality, mOsm/kg	278 ± 4 (270-299)
Na, meq/L	139.2 ± 1.7 (135-144)
K, meq/L	4.0 ± 0.3 (3.5-5.5)
Ca, mg/dL	9.4 ± 0.3 (8.0-11.3)
P, mg/dL	3.7 ± 0.6 (1.6-6.6)
Urine profile	
Creatinine, mg/dL	127.1 ± 84.0 (5-641)
Osmolality, mOsm/kg	649 ± 266 (59-1292)
sUCr/Osm	0.19 ± 0.08 (0.02-0.90)
UACR, mg/g	33.0 ± 323.0 (0.61-13333)

Abbreviations: CPK: creatine phosphokinase; GOT: glutamic oxaloacetic transaminase; GPT: glutamic pyruvic transaminase; MDRD eGFR: estimated glomerular filtration rate derived with Modification of Diet in Renal Disease (MDRD) Study equation; SD: standard deviation; sUCr/Osm: spot urine creatinine-to-osmolality ratio; UACR: urine albumin-to-creatinine ratio. To convert albumin to g/L, multiply by 10; Ca to mmol/L, 0.25; creatinine to *μ*mol/L, 88.4; glucose to mmol/L, 0.0555; P to mmol/L, 0.323; total bilirubin to *μ*mol/L, 17.1; urea nitrogen to mmol/L, 0.357; and uric acid to mmol/L, 0.059.

**Table 3:** Comparisons of spot urine creatinine according to conditions of demographics, comorbidities, and medication use.

	sUCr, mg/dL Mean ± SD	*P* value
Sex		
Male vs. female	143.2 ± 87.6 vs. 107.5 ± 75.0	<0.001
Races		
Mexican American	121.9 ± 73.6	<0.001^a^
Other Hispanic	118.2 ± 71.1	
Non-Hispanic White	115.5 ± 78.3	
Non-Hispanic Black	168.0 ± 95.0	
Non-Hispanic Asian	101.0 ± 72.7	
Other races	135.7 ± 84.3	
African American (yes vs. no)	168.0 ± 95.0 vs. 114.5 ± 76.1	<0.001
Comorbidities (yes vs. no)		
Hypertension	124.0 ± 76.1 vs. 128.2 ± 86.5	0.185
Diabetes mellitus	115.1 ± 66.2 vs. 128.7 ± 86.0	<0.001
Coronary artery disease	126.9 ± 69.6 vs. 127.1 ± 84.6	0.968
Congestive heart failure	126.0 ± 65.1 vs. 127.1 ± 84.4	0.911
Cerebrovascular disease	114.8 ± 85.0 vs. 127.4 ± 84.0	0.217
Active liver disease	113.5 ± 77.7 vs. 127.4 ± 84.1	0.217
Cancer	106.2 ± 67.1 vs. 128.6 ± 84.9	<0.001
Medication use (yes vs. no)		
ACEI/ARB	122.8 ± 75.8 vs. 127.9 ± 85.3	0.186
*β*-Adrenergic blocker	119.5 ± 72.5 vs. 127.8 ± 84.9	0.085
Calcium channel blocker	132.3 ± 81.1 vs. 126.8 ± 84.2	0.358
Diuretics	116.6 ± 71.8 vs. 128.1 ± 85.0	0.012
Other antihypertensives	133.1 ± 68.8 vs. 127.0 ± 84.4	0.502

Abbreviations: ACEI: angiotensin converting enzyme inhibitor; ARB: angiotensin II receptor blocker; sUCr: spot urine creatinine concentration. To convert creatinine to *μ*mol/L, multiplied by 88.4. ^a^As indicated, a comparison for the means among the various ethnic groups is performed using the one-way analysis of variance (ANOVA) along with the Bonferroni method as the post hoc test.

**Table 4:** Comparisons of spot urine osmolality according to conditions of demographics, comorbidities, and medication use.

	sUOsm, mOsm/kg Mean ± SD	*P* value
Sex		
Male vs. female	682.1 ± 256.9 vs. 609.0 ± 270.4	<0.001
Races		
Mexican American	694.5 ± 261.9	<0.001^a^
Other Hispanic	655.7 ± 268.9	
Non-Hispanic White	611.9 ± 261.6	
Non-Hispanic Black	709.9 ± 253.8	
Non-Hispanic Asian	600.5 ± 269.9	
Other races	682.5 ± 259.9	
African American (yes vs. no)	709.9 ± 253.8 vs. 630.5 ± 266.3	<0.001
Comorbidities (yes vs. no)		
Hypertension	622.8 ± 223.2 vs. 658.1 ± 277.8	<0.001
Diabetes mellitus	627.1 ± 222.6 vs. 652.1 ± 270.7	0.043
Coronary artery disease	631.8 ± 214.8 vs. 650.0 ± 267.7	0.325
Congestive heart failure	612.1 ± 227.2 vs. 649.9 ± 266.2	0.262
Cerebrovascular disease	566.2 ± 247.4 vs. 651.3 ± 265.7	0.004
Active liver disease	621.7 ± 249.9 vs. 649.7 ± 265.8	0.430
Cancer	576.8 ± 231.7 vs. 654.2 ± 267.0	<0.001
Medication use (yes vs. no)		
ACEI/ARB	617.1 ± 214.7 vs. 654.6 ± 272.8	0.001
*β*-Adrenergic blocker	582.3 ± 211.1 vs. 654.8 ± 268.9	<0.001
Calcium channel blocker	597.5 ± 192.8 vs. 652.7 ± 269.4	<0.001
Diuretics	588.4 ± 212.6 vs. 654.8 ± 269.2	<0.001
Other antihypertensives	607.5 ± 192.8 vs. 650.3 ± 267.2	0.046

Abbreviations: ACEI: angiotensin converting enzyme inhibitor; ARB: angiotensin II receptor blocker; sOsm: spot urine osmolality. ^a^As indicated, a comparison for the means among the various ethnic groups is performed using the one-way analysis of variance (ANOVA) along with the Bonferroni method as the post hoc test.

**Table 5:** Comparisons of spot urine creatinine-to-osmolality ratio according to conditions of demographics, comorbidities, and medication use.

	sUCr/Osm Mean ± SD	*P* value
Sex		
Male vs. female	0.20 ± 0.09 vs. 0.17 ± 0.08	<0.001
Races		
Mexican American	0.17 ± 0.07	<0.001^a^
Other Hispanic	0.17 ± 0.07	
Non-Hispanic White	0.18 ± 0.08	
Non-Hispanic Black	0.23 ± 0.10	
Non-Hispanic Asian	0.16 ± 0.07	
Other races	0.19 ± 0.08	
African American (yes vs. no)	0.23 ± 0.10 vs. 0.18 ± 0.07	<0.001
Comorbidities (yes vs. no)		
Hypertension	0.19 ± 0.09 vs. 0.19 ± 0.08	0.054
Diabetes mellitus	0.18 ± 0.09 vs. 0.19 ± 0.08	0.123
Coronary artery disease	0.20 ± 0.08 vs. 0.19 ± 0.08	0.199
Congestive heart failure	0.21 ± 0.09 vs. 0.19 ± 0.08	0.104
Cerebrovascular disease	0.20 ± 0.11 vs. 0.19 ± 0.08	0.305
Active liver disease	0.18 ± 0.09 vs. 0.19 ± 0.08	0.240
Cancer	0.18 ± 0.08 vs. 0.19 ± 0.08	0.080
Medication use (yes vs. no)		
ACEI/ARB	0.19 ± 0.10 vs. 0.19 ± 0.08	0.186
*β*-Adrenergic blocker	0.20 ± 0.10 vs. 0.19 ± 0.08	0.027
Calcium channel blocker	0.22 ± 0.12 vs. 0.19 ± 0.08	<0.001
Diuretics	0.20 ± 0.10 vs. 0.19 ± 0.08	0.315
Other antihypertensives	0.22 ± 0.10 vs. 0.19 ± 0.08	0.002

Abbreviations: ACEI: angiotensin converting enzyme inhibitor; ARB: angiotensin II receptor blocker; sUCr/Osm: spot urine creatinine-to-osmolality ratio. ^a^As indicated, a comparison for the means among the various ethnic groups is performed using the one-way analysis of variance (ANOVA) along with the Bonferroni method as the post hoc test.

**Table 6:** Standardized (*β*) coefficients of univariable linear regression models^a^ for spot urine profile.

	sUCr	*P*	sUOsm	*P*	sUCr/Osm	*P*
Age	-0.152	<0.001	-0.158	<0.001	-0.067	<0.001
Body weight	0.267	<0.001	0.173	<0.001	0.247	<0.001
Body mass index	0.151	<0.001	0.122	<0.001	0.109	<0.001
Blood biochemistry						
Urea nitrogen	0.084	<0.001	0.193	<0.001	-0.092	<0.001
Creatinine	0.259	<0.001	0.062	<0.001	0.365	<0.001
MDRD eGFR	-0.043	0.013	0.088	<0.001	-0.180	<0.001
Albumin	-0.045	0.009	-0.052	0.003	-0.014	0.428
GOT	0.027	0.121	-0.006	0.724	0.053	0.002
GPT	0.041	0.017	0.040	0.022	0.022	0.201
Total bilirubin	0.084	<0.001	0.028	0.111	0.109	<0.001
Uric acid	0.185	<0.001	0.070	<0.001	0.230	<0.001
CPK	0.200	<0.001	0.121	<0.001	0.193	<0.001
Glucose	0.029	0.097	0.066	<0.001	-0.031	0.070
Osmolality	0.090	<0.001	0.134	<0.001	-0.011	0.540
Na	0.069	<0.001	0.057	0.001	0.048	0.005
K	0.006	0.738	0.017	0.322	-0.011	0.527
Ca	-0.025	0.153	-0.050	0.004	0.018	0.295
P	-0.054	0.002	-0.038	0.031	-0.047	0.007
Urine profile						
sUCr	NA	NA-	0.815	<0.001	0.712	<0.001
sUOsm	0.815	<0.001	NA	NA	0.173	<0.001
sUCr/Osm	0.712	<0.001	0.173	<0.001	NA	NA
UACR	-0.056	0.001	-0.046	0.008	-0.039	0.024

Abbreviations: CPK: creatine phosphokinase; MDRD eGFR: estimated glomerular filtration rate derived with Modification of Diet in Renal Disease (MDRD) Study equation; GOT: glutamic oxaloacetic transaminase; GPT: glutamic pyruvic transaminase; NA: nonapplicable; sUCr: spot urine creatinine; sUCr/Osm: spot urine creatinine-to-osmolality ratio; sUOsm: spot urine osmolality; UACR: urine albumin-to-creatinine ratio. ^a^Performed with natural logarithm-transformed data of all variables.

**Table 7:** Standardized (*β*) coefficients of multivariable linear regression models^a^ for spot urine profile.

	sUCr	*P*	sUOsm	*P*	sUCr/Osm	*P*
Age	-0.010	0.303	-0.088	<0.001	-0.070	<0.001
Sex (ref. male)	0.027	0.018	-0.059	<0.001	0.029	0.147
Body weight	0.053	<0.001	-0.002	0.848	0.110	<0.001
African American (ref. non-African American)	0.068	<0.001	-0.010	0.249	0.151	<0.001
Comorbidities (ref. no presence)						
Hypertension	-^b^	-^b^	-^b^	-^b^	-^b^	-^b^
Diabetes mellitus	-^b^	-^b^	-^b^	-^b^	-0.038	0.017
CVA	-^b^	-^b^	-^b^	-^b^	-^b^	-^b^
Cancer	-^b^	-^b^	-^b^	-^b^	-^b^	-^b^
Medication use (ref. no user)						
*β*-Adrenergic blocker	-^b^	-^b^	0.022	0.019	-^b^	-^b^
Calcium channel blocker	0.020	0.029	-^b^	-^b^	0.034	0.031
Other antihypertensives	-^b^	-^b^	-^b^	-^b^	-^b^	-^b^
Blood biochemistry						
Urea nitrogen	-0.181	<0.001	0.250	<0.001	-0.247	<0.001
Creatinine	0.243	<0.001	-0.264	<0.001	0.384	<0.001
Albumin	-^b^	-^b^	-0.032	0.001	-^b^	-^b^
GOT	-^b^	-^b^	-^b^	-^b^	-^b^	-^b^
Total bilirubin	0.020	0.033	-^b^	-^b^	0.037	0.021
Uric acid	0.055	<0.001	-0.051	<0.001	0.095	<0.001
CPK	-^b^	-^b^	-^b^	-^b^	-^b^	-^b^
Glucose	-0.031	0.001	0.038	<0.001	-^b^	-^b^
Osmolality	-^b^	-^b^	0.037	0.001	-^b^	-^b^
Na	-^b^	-^b^	-^b^	-^b^	-^b^	-^b^
Ca	-^b^	-^b^	-^b^	-^b^	-0.037	0.016
P	-^b^	-^b^	-0.026	0.005	-^b^	-^b^
Urine profile						
sUCr	NA	NA	0.841	<0.001	NA	NA
sUOsm	0.818	<0.001	NA	NA	NA	NA
UACR	-^b^	-^b^	-^b^	-^b^	-^b^	-^b^
Adjusted *R*-square	0.752		0.744		0.257	

Abbreviations: CPK: creatine phosphokinase; CVA: cerebrovascular accident; GOT: glutamic oxaloacetic transaminase; NA: nonapplicable; ref.: reference group; sUCr: spot urine creatinine; sUCr/Osm: spot urine creatinine-to-osmolality ratio; sUOsm: spot urine osmolality; UACR: urine albumin-to-creatinine ratio. ^a^Performed with natural logarithm-transformed data of all variables. ^b^Omitted for conciseness, as the *P* value ≥ 0.05.

**Table 8:** Special conditions about interpretation of spot urine creatinine-to-osmolality ratio (sUCr/Osm).

sUCr/Osm tends to underestimate instant urinary excretion rate when:
Lower-than-ordinary Cr excretion load: muscle wasting, obesity, fluid retention (edema or ascites), chronic liver disease, and vegetarians
Decreased tubular Cr excretion: trimethoprim, cimetidine, and famotidine
Increased extrarenal Cr elimination: chronic kidney disease stage 5
Higher daily osmolar load: glycosuria in poorly controlled DM or taking SGLT-2i, extraordinarily large meals, and recent diuretics use

sUCr/Osm tends to overestimate instant urinary excretion rate when:
Higher-than-ordinary Cr excretion load: cooked meats, and creatine supplements
Increased tubular Cr excretion: nephrotic syndrome
Significant extrarenal osmolar loss: diarrhea, vomiting, gastric juice drainage, and excessive sweating

Abbreviations: Cr: creatinine; DM: diabetes mellitus; SGLT-2i: sodium-glucose cotransporter-2 inhibitors.

## Data Availability

The deidentified participant-level data of the 9,756 individuals enrolled in the NHANES 2011–2012 were first openly published in September 2013 and last revised in January 2015 (https://wwwn.cdc.gov/Nchs/Nhanes). The data sets with specified filtrations used in this study are publicly available and can be obtained on request. In addition, data dictionaries will be shared by the corresponding author on request.
